# Severe Neurologic Disorders in 2 Fetuses with Zika Virus Infection, Colombia

**DOI:** 10.3201/eid2306.161702

**Published:** 2017-06

**Authors:** Jorge Acosta-Reyes, Edgar Navarro, Maria José Herrera, Eloina Goenaga, Martha L. Ospina, Edgar Parra, Marcela Mercado, Pablo Chaparro, Mauricio Beltran, Maria Luz Gunturiz, Lissethe Pardo, Catalina Valencia, Sandra Huertas, Jorge Rodríguez, Germán Ruiz, Diana Valencia, Lisa B. Haddad, Sarah C. Tinker, Cynthia A. Moore, Hernando Baquero

**Affiliations:** Universidad del Norte, Barranquilla, Colombia (J. Acosta-Reyes, E. Navarro, M.J. Herrera, H. Baquero);; Secretaría de Salud de Barranquilla, Barranquilla (E. Goenaga);; Instituto Nacional de Salud, Bogotá, Colombia (M.L. Ospina, E. Parra, M. Mercado, P. Chaparro, M. Beltrán, M.L. Gunturiz, L. Pardo);; Clínica Colsanitas, Clínica Reina Sofía, Bogotá (C. Valencia, S. Huertas, J. Rodríguez, G. Ruiz);; Centers for Disease Control and Prevention, Atlanta, Georgia, USA (D. Valencia, S.C. Tinker, C.A. Moore);; Emory University School of Medicine, Atlanta (L.B. Haddad).

**Keywords:** Zika virus, congenital Zika virus infection, pediatric infections, neurologic disorders, viruses, Colombia

## Abstract

We report the results of pathologic examinations of 2 fetuses from women in Colombia with Zika virus infection during pregnancy that revealed severe central nervous system defects and potential associated abnormalities of the eye, spleen, and placenta. Amniotic fluid and tissues from multiple fetal organs tested positive for Zika virus.

In October 2015, Colombia confirmed its first case of autochthonous Zika virus infection (*1*). As of November 2016, ≈105,000 cases of symptomatic Zika virus disease have been reported, including 19,499 cases among pregnant women. Although Zika virus infections typically lead to comparatively benign symptoms relative to those of other arboviruses (*2*), in May 2016, the US Centers for Disease Control and Prevention concluded that congenital Zika virus infection was the cause of the severe central nervous system (CNS) defects observed in fetuses and newborns of women infected with Zika virus during pregnancy (*3*). Although the full spectrum of fetal effects of congenital Zika virus infection is not known, Zika virus has been shown to cross the placental barrier, grow in brain tissue of fetuses, infect progenitor neural cells, and increase neural cell death or attenuate their growth (*4*,*5*). Observed CNS abnormalities include microcephaly, ventriculomegaly, cerebral calcifications, absent corpus callosum, and atrophy of the cerebellum and brainstem in fetuses with congenital Zika virus infection (*6*). This report describes 2 fetuses examined after pregnancy termination who had severe CNS defects attributed to maternal Zika virus infection.

## The Cases

Case 1 involved a 24-year-old pregnant resident of the city of Barranquilla who had symptoms compatible with Zika virus disease, including fever and generalized rash for 2 days, followed by edema and joint pain for 2 weeks, with onset occurring at 5–6 weeks of gestation. Her medical history was notable because of a prior pregnancy complicated by a stillbirth with Potter syndrome, delivered 2 months before conception of the current pregnancy. Obstetric ultrasounds at 8 and 12 weeks of gestation showed a singleton fetus with no abnormalities; an ultrasound at 15 weeks of gestation demonstrated absence of the cranial vault (exencephaly) with brain tissue loss, including absence of cerebral hemispheres (anencephaly sequence). Amniotic fluid sampled at 17 weeks was positive for Zika virus by real-time reverse transcription PCR (rRT-PCR). The fetus was at 20 weeks of gestation when evaluated.

Case 2 involved a 15-year-old pregnant resident of Bogotá who reported symptoms compatible with Zika virus disease, including generalized rash for 2 days and myalgia, after travel to a municipality with prevalent Zika virus infections, with onset occurring at 16–20 weeks of gestation. She had an uncomplicated obstetric history with 1 prior live birth. Prenatal care started in week 26; an ultrasound at that time showed a fetus with abnormal clefts in the cerebral hemispheres of the brain (schizencephaly), with cleft walls separated and filled with cerebrospinal fluid (open lip). Follow-up ultrasound indicated large regions of parietal and temporal brain parenchymal loss. The fetus was evaluated and Zika virus diagnosis confirmed at 27 weeks of gestation.

Autopsies of fetal organs and placenta with anatomic pathology and microscopic evaluation were performed for both fetuses. Tissue samples were fixed in 10% neutral-buffered formalin and fixed in paraffin; 4-μm-thick cuts were stained with hematoxylin and eosin for morphologic analysis with light microscopy. RNA was extracted from fresh tissue by using a TRIzol plus RNA purification kit (Thermo Fisher Scientific, Waltham, MA, USA). We used rRT-PCR for the detection of Zika virus (NS5) and 1-step RT-PCR for the detection of the envelope protein–coding region (360 bp) were performed as previously described (*7*,*8*). Immunohistochemical markers were used in tissue from both fetuses. Molecular testing for syphilis, toxoplasmosis, rubella, and cytomegalovirus was performed following techniques described previously (*9*,*10*); 500-band resolution karyotype was also performed for both fetuses, and ELISA testing for HIV was performed on maternal serum samples.

We documented notable abnormalities in the CNS, eye, spleen, and placenta for 1 or both fetuses (Table). All tissues tested and amniotic fluid were positive for Zika virus except eye and placenta from the fetus in case 2. Immunohistochemical markers were negative. For both cases, all tissue samples were negative for other congenital infections (i.e., syphilis, toxoplasmosis, rubella, and cytomegalovirus), high-resolution karyotype was normal, and maternal serum was HIV-negative.

**Table:** Summary of 2 case reports involving fetuses examined after pregnancy termination who had severe neurologic defects attributed to maternal Zika virus infection, Colombia*

Characteristics	Case 1	Case 2
Pregnancy history		
Maternal age, y	24	15
Municipality of residence	Barranquilla, Colombia	Bogotá, Colombia; recent travel to area with circulating Zika virus
Gestational age at maternal infection, wks	5–6	16–20
Maternal symptoms	Fever; generalized rash for 2 d; edema and joint pain for 2 wks	Generalized rash for 2 d and myalgia
Prior pregnancy complications	Previous pregnancy complicated by Potter syndrome ending in stillbirth 2 mo before current pregnancy	None; one previous live birth
Plurality	Singleton	Singleton
Ultrasound findings	8 wks: no notable abnormalities; 12 wks: no notable abnormalities; 15 wks: absence of cranial vault with brain tissue loss including absence of cerebral hemispheres (exencephaly–anencephaly sequence)	26 wks: abnormal clefts in cerebral hemispheres of the brain (schizencephaly) with open lip; 26 wks (4 d later): large regions of parietal and temporal brain parenchyma loss
Samples obtained	Amniotic fluid from amniocentesis (17 wks); cord blood from amniocentesis (17 wks); tissue samples of fetal organs (placenta, liver, kidney, spleen, cord, eyes, brain tissue, spinal cord)	Amniotic fluid from amniocentesis (27 wks); cord blood from amniocentesis (27 wks); tissue samples of fetal organs (placenta, liver, kidney, spleen, cord, eyes, brain tissue, spinal cord)
Pregnancy outcome	Elective termination at 20 wks	Elective termination at 27 wks
Macroscopic and histopathologic examination		
CNS	Anencephaly, without associated malformations; absence of cerebral hemispheres and cerebellum; rudimentary central trunk; absence of bones of the upper calvaria, with purplish brown irregular tissue attached to the base corresponding to residual nerve tissue; recent bleeding in tissues of the cortex and brain stem; decreased neuroblast density compatible with neuronal migration arrest and glial leptomeningeal heterotopy	Almost complete absence of periventricular germinal matrix of CNS tissue, with replacement by reactive glia; decreased pattern of maturation/migration of cerebral cortex with edema and congestion; neuropil disruption with microcalcification in the spinal cord, with reactive gliosis and loss of the ascending and descending tracts of the spinal cord, with congestion; peripheral nerve ganglion of the sympathetic chain with Wallerian degeneration and reactive proliferation of sustentacular cells, satellitosis, and neuronophagia of ganglion cells, and neuronal degeneration
Eye	Neuroepithelium of the retina without disruptive changes or microcalcifications	Neuroepithelium of the retina not identified
Spleen	Congestion; adequate development of white and red pulp	Complete autolysis
Chorionic villi	Immature chorionic villi with enlarged irregular contours without inflammatory infiltrate and partial obliteration of fetal vessels	Heterogeneous pattern of accelerated maturation of the chorionic villi, with some chorionic villi enlarged with irregular surroundings with irregular growth pattern
Umbilical cord	Unremarkable	Single umbilical artery
Placenta	Slight increase in perivillous fibrin deposits; chronic lymphocytic deciduitis	Increase in perivillous fibrin deposits
Kidney	Unremarkable	Unremarkable
Liver	Unremarkable	Unremarkable
Thymus	Not reported	Unremarkable
Lung	Not reported	Unremarkable
RT-PCR results for Zika virus in fetal tissues		
Brain	Positive	Positive
Brain stem	Positive	Not reported
Spinal cord	Positive	Not reported
Eye	Positive	Negative
Spleen	Positive	Positive
Kidney	Positive	Positive
Liver	Positive	Positive
Thymus	Not reported	Positive
Lung	Not reported	Positive
Myocardium	Not reported	Positive
Bone marrow	Not reported	Positive
Peripheral nerve	Not reported	Positive
Umbilical cord	Positive	Positive
Placenta	Positive	Negative

*CNS, central nervous system; RT-PCR, reverse transcription PCR.

**Figure 1:**
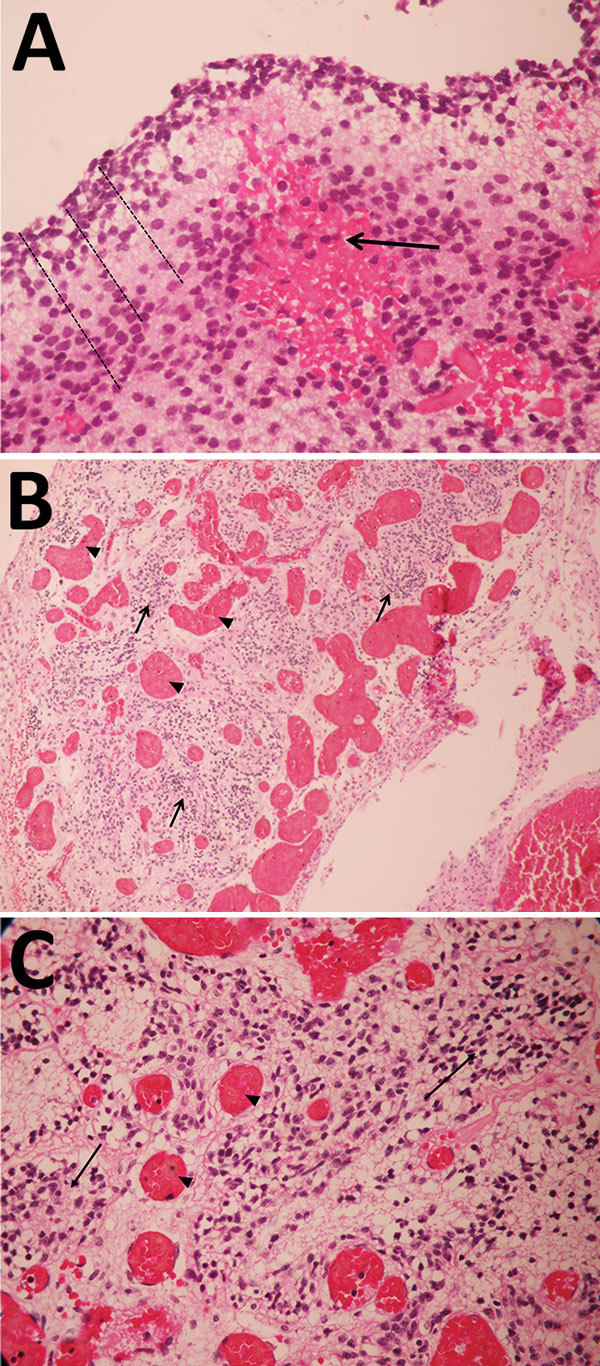
Pathology findings for case 1, involving a fetus examined after pregnancy termination who had severe neurologic defects attributed to maternal Zika virus infection, Colombia. A) Remnant tissue of cerebral cortex showing a reduced neuroblast layer (dotted lines) and hemorrhagic foci (arrow). Hematoxylin and eosin (H&E) staining; original magnification ×40. B) Glial leptomeningeal heterotopy showing congestive blood vessels (arrowhead) and foci of glial heterotopia (arrows). H&E staining; original magnification ×10. C) Glial leptomeningeal heterotopy showing congestive blood vessels (arrowhead) and foci of glial heterotopia (arrows). H&E staining; original magnification ×40.

**Figure 2:**
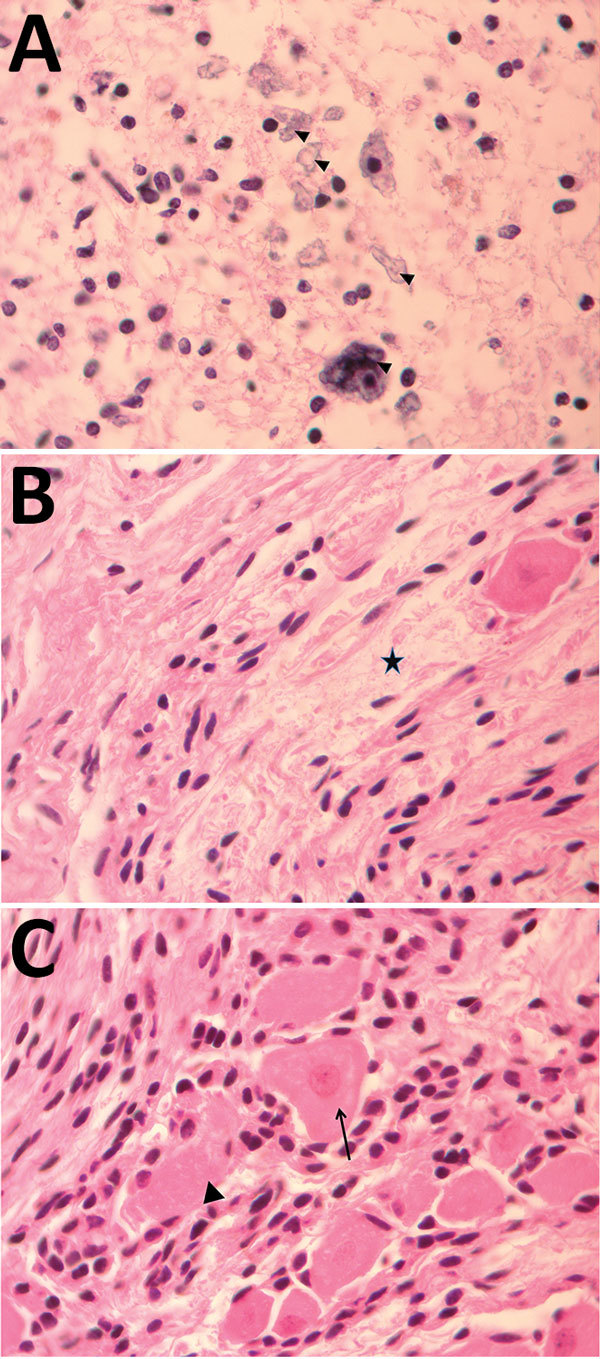
Pathology findings for case 2, involving a fetus examined after pregnancy termination who had severe neurologic defects attributed to maternal Zika virus infection, Colombia. A) Spinal cord slice showing neuropil disruption with multiple calcifications (arrowheads). B) Nerve showing disruptive changes of axons (Wallerian degeneration) (black star). C) Dorsal root ganglion showing spinal ganglion with satellitosis (arrow) and neuronophagia of ganglion cells (arrowhead). Hematoxylin and eosin staining; original magnification ×100.

For case 1, microscopic examination demonstrated evidence of recent bleeding in the residual tissues of the cerebrum and brain stem, with no microcalcifications, inclusion bodies, or inflammatory infiltrate (Figure 1). For case 2, we observed microcalcifications in areas of neurologic degeneration; a spinal cord slice showed neuropil disruption with multiple calcifications, reactive gliosis, and loss of the ascending and descending spinal cord tracts (Figure 2).

These 2 cases of fetal Zika virus infection involved severe brain tissue damage and expand the spectrum of CNS defects that might result from congenital Zika virus infection (*11*). Multiple tissues tested positive for Zika virus in both cases, providing strong evidence of vertical transmission. For case 2, the observed pattern of arrest in neuronal migration and decrease in the pattern of maturation of the CNS is consistent with the potential effects of Zika virus infection noted in previous reports (*5*). For case 1, although Zika virus was demonstrated in multiple tissues of the fetus, the birth defect identified (anencephaly) is not on the recognized spectrum of congenital infection. The timing of exposure and primary affected tissue type potentially implicate Zika virus in disruption of neural tube closure in this case. However, the pathology evaluation was consistent with expected findings in any fetus with anencephaly; therefore, congenital Zika virus might have been coincidental in a fetus already predestined by other factors to have anencephaly.

The identification of virus in tissues outside of the brain suggests the need to consider other organs as targets for the virus. To improve knowledge of the spectrum of outcomes among pregnancies affected by congenital Zika virus and to identify interventions that might reduce illness among affected newborns, such as hearing and visual screening, it will be necessary to look for damage in organs other than the brain among newborns of mothers with Zika virus infection during pregnancy. The mothers of the fetuses in these 2 cases reported Zika virus disease symptoms at different times during the pregnancy (early first trimester and early second trimester), which highlights the potentially long window of vulnerability to adverse outcomes among pregnant women with Zika virus infection.

Persistent viremia in maternal serum has been previously reported (*12**–**14*); we observed positive results for Zika virus molecular tests performed on fetal tissues and amniotic fluid 15 week (case 1) and 10 weeks (case 2) after symptoms of acute infection in the pregnant women. Although we did not test for replication-competent virus, the virus probably persists in different fetal tissues after primary infection because of replication in those tissues. The virus might also replicate in placental tissue (*12*,*15*), although it is probably not the only location for viral replication in the 2 cases described in this report, given that Zika virus was identified in the placenta for only 1 of the cases.

Given the epidemic levels of Zika virus disease being reported in Colombia and the severe outcomes associated with congenital infection, we call attention to the importance of healthcare providers reporting cases of Zika virus disease for adequate public health surveillance. Recommendations might include enhanced surveillance of acute cases among pregnant women and stillbirths and abortions related to Zika virus disease during pregnancy, as well as adverse outcomes among live births. Collecting information on congenital Zika virus infection is important to helping public health authorities effectively address this new health threat and make evidence-based decisions, which will benefit Colombia and other countries throughout the world.
